# Prevalence of metabolic syndrome in non-diabetic, pregnant Angolan women according to four diagnostic criteria and its effects on adverse perinatal outcomes

**DOI:** 10.1186/s13098-016-0139-3

**Published:** 2016-03-22

**Authors:** Hamilton dos Prazeres Tavares, Débora Cristina Damasceno Meirelles dos Santos, Joelcio Francisco Abbade, Carlos Antonio Negrato, Paulo Adão de Campos, Iracema Mattos Paranhos Calderon, Marilza Vieira Cunha Rudge

**Affiliations:** Department of Gynecology and Obstetrics, Botucatu Medical School, UNESP—Univ Estadual Paulista, Botucatu, São Paulo Brazil; Department of Gynecology and Obstetrics, Medical School, University Agostinho Neto (UAN), Luanda, Angola

**Keywords:** Prevalence, Metabolic syndrome, Angola, Pregnancy perinatal outcome

## Abstract

**Background:**

Metabolic syndrome (MetS) is a cluster of risk factors for type 2 diabetes (Type2 DM) and cardiovascular diseases (CVD), and its prevalence varies based on region, population, and sex. Newborns of women with MetS have a greater risk of adverse perinatal outcomes. This study explores the prevalence of metabolic syndrome in non-diabetic, pregnant Angolan women and the adverse perinatal outcomes associated with it.

**Methods:**

This cross-sectional study collected the demographic, anthropometric and clinical data of 675 pregnant women in the maternity ward of General Hospital in Huambo, Angola. Metabolic syndrome was defined using four criteria: the third report of the National Cholesterol Education Program Adult Treatment Panel (ATPIII), the Joint Interim Statement (JIS), and definitions by both Bartha et al. and Chatzi et al.

**Results:**

The crude prevalence of metabolic syndrome was 36.6 % based on the JIS definition, 29.2 % based on NCEP ATPIII, 12.6 % based on Chatzi et al. and 1.8 % based on Bartha et al. In general, the prevalence of adverse perinatal outcomes was 14.1 %.

**Conclusions:**

There was a high prevalence of metabolic syndrome, depending on the criteria used, and thus a great need to harmonize the criteria and cutoff points. Perinatal adverse outcomes were higher in pregnant women with metabolic syndrome.

## Background

Metabolic syndrome (MetS) is a cluster of risk factors for type 2 diabetes (Type2 DM) and cardiovascular diseases (CVD), with insulin resistance (IR) proposed as the linking factor [1]. It includes dyslipidemia elevated triglycerides and apolipoprotein B (apoB), containing lipoproteins and low high-density lipoproteins (HDL), as well as elevated arterial blood pressure (BP) and dysregulated glucose homeostasis. Abdominal obesity and/or IR have also gained increasing attention as important components of the syndrome. Despite the many components and clinical implications of MetS, there are still no universally accepted pathogenic mechanisms or clearly defined diagnostic criteria [2], for the syndrome.

Over the past few decades, several definitions of MetS have been proposed using various diagnostic criteria: WHO [3, 4], the National Cholesterol Education Program Third Adult Treatment Panel (ATPIII) [5], and more recently, the International Diabetes Federation (IDF) (2005). In an attempt to unify the criteria, in 2009, an additional definition was proposed as a harmonized Joint Interim Statement (JIS) by several organizations. Notwithstanding the various definitions, central obesity, dyslipidemia, hyperglycemia and elevated BP are the key characteristics of MetS [6].

In the same year as the JIS publication, a study conducted in Brazil showed that the prevalence of MetS in pregnancy increases with the decline in glucose tolerance. The study also demonstrated that the glycemic profile is a useful diagnostic tool for identifying metabolic abnormalities related to MetS in pregnancy and predicting the occurrence of adverse perinatal outcomes (APO). The authors concluded that MetS diagnosed during mid-pregnancy is a predictor of APO, both in women with gestational diabetes (GDM) and in those with mild gestational hyperglycemia (MGH) who are not currently classified as having GDM. These results reinforced the importance of screening for the components of MetS to identify pregnant women who are at high risk for APO [7].

During the same period, research was published studying different populations and showed an association between maternal MetS in early pregnancy and higher risk for both preterm birth [8] and GDM [9].

In 2008, Bartha et al. modified the NCEP ATPIII for pregnancy, establishing a new cutoff for several parameters: pre-pregnancy BMI >30 kg/m^2^, plasma triglycerides ≥2 SD for gestational age, HDL cholesterol >2 SD for gestational age, blood pressure ≥130/≥85 mm/Hg, and fasting glucose ≥110 mg/dl [10].

Recognition of MetS during pregnancy could help identify a subgroup of women who may not only develop a pregnancy-related condition but are also potentially at an increased risk for either metabolic or cardiovascular adverse conditions later in life.

Adverse outcome indexes were designed to measure the quality of perinatal care [11–13].

The clinical outcomes are a function of both the received healthcare and the basic health status of the patient [15].

In sub-Saharan Africa, many countries are experiencing a rapid demographic and epidemiological transition [16].

Angola is a country in sub-Saharan Africa that has undergone significant political changes over the last several years, accompanied by rapid economic growth and an increased urbanization rate. These changes may be implicated in the increasing prevalence of MetS caused by the rise in obesity due to insufficient physical activity, dyslipidemia, hypertension and hyperglycemia [16]. However, the prevalence of the MetS in pregnant Angolan women, the specific factors contributing to its occurrence and its perinatal implications remain unknown. Despite the diversity of definitions, the prevalence of MetS is well known in several populations worldwide [2]. To date, however, no such information is available in communities in low- to mid-income countries, such as non-diabetic, pregnant Angolan women. There are also no studies on the association between APO and maternal MetS in pregnancy.

Our hypothesis is that in a country with a high prevalence of MetS, norm glycemic pregnant women may also exhibit a high prevalence of MetS, which has direct effects on the perinatal results. The detection of MetS during pregnancy has not been established in the literature, and it merits a comparison between different diagnostic criteria.

This study aimed to first-gather data on the prevalence of MetS in a subset of non-diabetic, pregnant Angolan women; second-estimate the prevalence of MetS according to four diagnostic criteria: NCEP ATP III [5], the Joint Interim Statement (JIS) definition [6], and the modification of criteria for pregnancy proposed by Bartha et al. [10] and Chatzi et al. [8, 9]; first-determine the level of agreement and disparity among these four criteria for the diagnosis of MetS and APO; and forth-compare the effects of MetS in non-diabetic, pregnant women on APO.

## Methods

### Study population

This mother–child, cross-sectional study examined a sample population of non-diabetic pregnant women from the public General Hospital of Huambo, Angola, to estimate the prevalence of metabolic syndrome (MetS) in pregnancy and its effects on APO. During the study period, which lasted from December 2014 to February 2015, a total of 675 single, non-diabetic pregnant women with complete data were selected and enrolled in this study.

The study was conducted according to the tenets of the Declaration of Helsinki, and the protocol was approved by the Ethics Committee of the Faculty of Medicine, Agostinho Neto University, Huambo, Angola.

All non-diabetic pregnant women in the maternity ward during this period were invited to participate and were included after an informed consent form was signed. Patients with established cardiovascular diseases, thyroid dysfunction, excessive alcohol or other drug abuse, current or recent psychiatric treatment (upto 4 months) during pregnancy and cesarean section were excluded. Eight nurses were trained on the structured guide for the survey. The interview guide was divided into three sections: socio-demographic characteristics, anthropometrics and biochemical evaluation.

On the test day, all consenting subjects provided information for a structured questionnaire, underwent an anthropometric examination and provided blood samples for biochemistry tests. Maternal characteristics such as age, parity, ethnicity, educational level, family income, smoking status, alcohol consumption and alimentary habits, weight and pre-pregnancy body mass index (BMI) were recorded.

All biochemical analyses were carried out within 2 h of blood sampling in the laboratory of the General Hospital of Huambo.

Pre-gestational BMI and waist circumference (WC) were obtained to quantify obesity. Body weight (kg) was measured to the nearest 0.1 kg using a previously calibrated mechanical scale (SECA GmbH & Co, Germany) with a maximum capacity of 200 kg. Standing body height (cm) was measured to the nearest 0.2 cm using a portable wall stadiometer (Seca, Germany). WC was measured in cm at the level of the navel using a flexible, non-distensible tape without exerting pressure on the tissues. Pre-gestational BMI was calculated as weight in kilograms divided by the square of the height (kg/m^2^). Three consecutive measurements of sitting blood pressure at a minimum of 5-min intervals were recorded using Omrom^®^ MX3 with an automated oscillometric Blood Pressure Monitor (O-HEM-742-E) (Matsusaka, Japan) [17].

After the participants had been sitting for at least 30 min, were measures Systolic blood pressure (SBP, mmHg) and diastolic blood pressure (DBP, mmHg). Hypertension was defined as systolic blood pressure ≥130 mmHg and diastolic blood pressure ≥85 mmHg.

In addition, venous blood samples were drawn from the forearm using standard techniques and were processed immediately using commercially available kits (BioSystems SA, Costa Brava 30, Barcelona, Spain) to measure fasting plasma glucose (FPG, mg/dl), triglycerides (TG, mg/dl), total cholesterol (TC, mg/dl), low-density lipoprotein cholesterol (LDL-C, mg/dl), and high-density lipoprotein cholesterol (HDL-C, mg/dl). All biochemical analyses were carried out within 2 h of blood sampling in the central laboratory of Huambo Hospital. This lab adheres to strict internal and external standards of quality control techniques.

### Definition of metabolic syndrome in pregnancy

All participants were classified as either having or not having MetS according to four criteria, as indicated in Table 1, NCPE ATP III 2001 [5], JIS and two modified definitions for pregnancy: Bartha et al. [10] and Chatzi et al. [8, 9].

**Table 1:** Criteria for clinical diagnosis of metabolic syndrome components according to four definitions

Components of MetS	NCEP–ATP III [5]	JIS MetS Criteria [6]	NCEP–ATP III, Bartha et al. [10] modification	HNLBI/AHA, Chatzi et al. [8, 9] modification
Prerequisite	None	None	None	None
No of other criteria	≥3 of:	≥3 of:	≥3 of:	≥3 of:
Obesity	WC >88 cm	WC ≥ 80 cm	PG/BMI >30 kg/m^2^	PG/BMI >30 kg/m^2^
Hypertension (mm/Hg)	≥130/85	≥130/85	≥130/85	≥130/85
Decreased HDL-C	<50 mg/dl	<50 mg/dl	<2 SD (40 mg/dl)	<50 mg/dl
Hypertriglyceridemia	≥150 mg/dl	≥150 mg/dl	≥2 SD/GA (270 mg/dl)	≥150 mg/dl
Increased fasting hyperglycemia	≥110 mg/dl	≥100 mg/dl	≥105 mg/dl	≥100 mg/dl

The definition of the four criteria was based on the presence of three or more of the five components (Table 1).

The ATP III [5] components are WC >88 cm; SBP ≥130 mmHg and/or DBP ≥85 mmHg and/or BP-lowering treatment; fasting triglyceride levels ≥150 mg/dl and/or treatment for hypertriglyceridemia; HDL-C <50 mg/dl and/or treatment for dyslipidemia; and fasting glucose level ≥110 mg/dl and/or on antidiabetic medication.

The Joint Interim Statement (JIS) definition [6] components are WC >80 cm; SBP ≥130 mmHg and/or DBP ≥85 mmHg and/or undergoing BP-lowering treatment; fasting triglyceride levels ≥150 mg/dl and/or treatment for hypertriglyceridaemia; HDL-C <50 mg/dl and/or treatment for dyslipidemia; and fasting glucose level ≥100 mg/dl or on antidiabetic medication.

The Bartha et al. [10] modified criteria of NCEP APTIII characterize MetS by abdominal obesity, given WC >2 SD for gestational age in the first half of pregnancy or pre gestational BMI >30 kg/m^2^; triglycerides ≥2 SD for gestational age; HDL-cholesterol <2 SD for gestational age; blood pressure ≥130/85 mm/Hg; and fasting glucose ≥105 mg/dl.

The Chatzi et al. [8, 9] criteria, which modify HNLBI/AHA and NECP ATP III, are as follows: pre gestational BMI >30 kg/m^2^; triglycerides ≥150 mg/dl; HDL cholesterol <50 mg/dl; BP ≥130/85 mm/Hg; and fasting glucose ≥100 mg/dl.

### Adverse perinatal outcomes

Newborn data collection included birth weight, length, sex, gestational age at delivery, the first and fifth minutes’ perinatal morbidity and congenital malformations. Births were defined as pre-term if the gestational age was <37 weeks. The relation between the newborns’ weight and gestational age was defined according to Lubchenco’s criteria [18].

APO was diagnosed in the presence of any morbidity factors, such as prematurity, a low Apgar score, malformations, respiratory distress syndrome, jaundice, infections, large for gestational age (LGA), small for gestational age (SGA) and macrosomia.

### Statistical analysis

Data analyses were performed using the SAS 9.3 Foundation for Microsoft^®^ Windows^®^ (Copyright © 2012, SAS Institute Inc., Cary, NC, USA). Data for categorical variables are expressed as the number and percentage. Continuous variables are reported as the mean ± standard deviation and compared using the independent samples *t* test. Categorical variables were expressed as proportions and compared using the Chi square test or Fisher’s exact test if appropriate. The prevalence of MetS was determined according to the four criteria (NCEP ATP III 2001, the harmonized definition, the Bartha et al. criteria that modified NCEP APT III and the Chatzi et al. criteria that modified HNLBI/AHA). The kappa coefficient was calculated to evaluate the concordance among the definitions—NCEP ATP III, the harmonized criteria, Bartha et al. and Chatzi et al.—in detecting MetS and APO.

The agreement among the four definitions was determined by kappa statistics (κ). The level of agreement was considered poor for κ ≤ 0.20, fair for κ = 0.21–0.40, moderate for κ = 0.41–0.60, substantial for κ = 061–0.80 and excellent for κ ≥ 0.80 [19].

The percentage of socio-demographic and obstetric variables, the prevalence of MetS, and the APO of non-diabetic pregnant women with MetS were calculated and grouped as independent and dependent variables.

Independent variables: with or without MetS. Dependent variables: with or without APOs. Comparisons between ‘with’ and ‘without’ MetS were made using a difference of proportions test for all criteria. Sensibility, specificity, and positive and negative predicted values were also calculated for all of the criteria used to diagnose of MetS and for APO. A *p* value <0.05 was considered statistically significant.

## Results

A total of 675 non-diabetic pregnant women consented to participate in the survey. Full data sets were available from all participants and are the basis of this report. The mean values for the socio-demographic and various anthropometric, clinical and biochemical parameters of the study patients are presented in Table 2.

**Table 2:** Frequency analysis of socio-demographic and metabolic parameters of pregnant women

Variables	Media ± standard deviation
N	675
Race	
Black [n (%)]	675 (100)
Other [n (%)]	0 (0)
Maternal age (years)	24.7 ± 6.7
Level of education	
Never studied [n (%)]	54 (8)
Middle [n (%)]	572 (84.7)
Higher education [n (%)]	49 (7.3)
Place of residence	
Urban [n (%)]	149 (22.1)
Village [n (%)]	526 (77.9)
Occupation	
Students [n (%)]	141 (21.0)
Business [n (%)]	460 (68.1)
Public official [n (%)]	74 (11.0)
Smoking habit	
Non-smoker	674 (99.9)
Current smoker (%)	1 (0.1)
Gestational age (weeks)	39.2 ± 1.6
Hypertension [n (%)]	370 (54.8)
Anti-hypertensive drugs (%)	44 (6.5)
Nulliparous	188 (27.8 %)
Glycemic level (mg/dl)	
≥110	70 (10.4)
≥105	90 (13.3)
≥100	124 (18.4)
<100	551 (81.6)
Fasting glycemia mean	83.7 ± 22.9
Obesity	
Mean waist circumference	93.6 ± 9.3
BMI (kg/m^2^)	24.4 ± 4.2
Triglycerides (mg/dl)	149.3 ± 55.8
HDL-C (mg/dl)	70.9 ± 18.5
LDL-C (mg/dl)	116.9 ± 34.5
Total cholesterol (mg/dl)	193.2 ± 37.8
Adverse perinatal outcome (APO)	95 (14.1 %)

The mean age of the patients was 24 ± 6.7 years, and the mean gestational age was 39.2 weeks. In terms of educational background, 8.0 % were illiterate, 84.7 % had completed secondary education and 7.3 % had completed high school. One hundred forty-nine patients (22.1 %) were from urban areas, and 572 (84.7 %) were from rural areas. Only one patient was a smoker at the time of the study.

The major occupations of the patients were business (460, 68.1 %), student (141, 21.0 %) and public officials (74, 11.0 %). One hundred eighty-eight (27.8 %) were nulliparous.

In our study, the mean values of particular features of metabolic abnormalities characteristic of MetS among pregnant women are summarized in Table 2. Most of the women studied had normal blood glucose levels (551, 81.6 %). Obesity was evaluated by two parameters: the mean pre-gestational BMI was 24.4 ± 4.2, and the mean WC at term was 93.6 ± 9.3 cm. The mean fasting plasma glucose was 83.7 ± 22.9 mg/dl, and the triglyceride, HDL-C, LDL-C and total cholesterol means were 149.3 ± 55.8, 70.9 ± 18.5, 116.9 ± 34.5, 193.2 ± 37.8, respectively. Hypertension was very common among pregnant women and was detected in 54.8 % of all patients; 6.5 % were taking medication for hypertension.

APOs were diagnosed in 14.1 % of the entire population.

The prevalence of MetS was estimated using NCEP ATP III, the Joint Interim Statement (JIS) definition, and the modified criteria for MetS in pregnancy by Bartha et al. [10] and by Chatzi et al. [9]. The overall prevalence of MetS during pregnancy varied according to the four definitions: 36.6 % for the JIS, 29.2 % for the NCEP ATP III, 12.6 % for the Chatzi definition and 1.8 % for the Bartha definition. These results are shown in Table 3 (p < 0.0001).

**Table 3:** Prevalence of metabolic syndrome according to four definitions and its components in pregnant women

Components of MetS	NCEPT–ATP III [5] N = 675		JIS MetS Criteria [6] N = 675		NCEPT–ATP III Bartha [10] modification N = 675		NHLBI/AHA Chatzi [8, 9] modification N = 675	
	Cut points	N (%)	Cut points	N (%)	Cut points	N (%)	Cut points	N (%)
Fasting glycemia mean	≥110 mg/dl	70 (10.4)	≥100 mg/dl	124 (18.4)	≥105 mg/dl	90 (13.3)	≥100 mg/dl	124 (18.4)
Obesity								
BMI		–	–		>30 kg/m^2^	75 (11.1)	>30 kg/m^2^	75 (11.1)
Waist circumference	>88 cm	497 (73.6)	≥80 cm	657 (97.3)		_	–	–
Hypertriglyceridemia	≥150 mg/dl	293 (43.4)	≥150 mg/dl	293 (43.4)	≥270 mg/dl	15 (2.2)	≥150 mg/dl	293 (43.4)
Decreased HDL-C	<50 mg/dl	53 (7.8)	<50 mg/dl	53 (7.9)	<40 mg/dl	17 (2.5)	<50 mg/dl	53 (7.9)
Hypertension	≥130/85 mm/Hg	370 (54.8)	≥130/85 mm/Hg	370 (54.8)	≥ 130/85 mm/Hg	370 (54.8)	≥ 130/85 mm/Hg	370 (54.8)
MetS prevalence		197 (29.2)		247 (36.6)		12 (1.8)		85 (12.6)

* Differences of the test proporcções—p < 0.0001 (a, b, c and d)

The prevalence of individual MetS components according to the four criteria varied according to different criteria, and the results are presented in Table 4.

**Table 4:** Prevalence of individual metabolic syndrome abnormalities in pregnant women with metabolic syndrome according to four definitions

Components of MetS	NCEPT–ATP III [5] N = 197		JIS MetS Criteria [6] N = 247		NCEPT–ATP III Bartha [10] modification N = 12		NHLBI/AHA Chatzi [8, 9] modification N = 85	
Increased fasting glycemia	≥110 mg/dl	44 (22.3)	≥100 mg/dl	89 (36.0)	≥105 mg/dl	10 (83.3)	≥100 mg/dl	40 (47.1)
Obesity								
BMI					>30 kg/m^2^	11 (91.7)	>30 kg/m^2^	40 (47.1)
Waist circumference	>88 cm	189 (95.9)	≥80 cm	246 (99.6)		–		–
Hypertriglyceridemia	≥150 mg/dl	166 (84.3)	≥150 mg/dl	199 (80.6)	≥270 mg/dl	1 (8.3)	≥150 mg/dl	78 (91.8)
Decreased HDL-C	<50 mg/dl	45 (22.8)	<50 mg/dl	49 (19.8)	<40 mg/dl	2 (16.7)	<50 mg/dl	32 (37.6)
Hypertension	≥130/85 mm/Hg	179 (90.9)	≥130/85 mm/Hg	216 (87.4)	≥130/85 mm/Hg	12 (100)	≥130/85 mm/Hg	79 (92.9)
Metabolic syndrome %		29.2 (a)		36.6 (b)		1.8 (c)		12.6 (d)

Using the JIS definition, 36.0 % had high fasting blood glucose levels, 99.6 % had increased WC, 80.6 % had hypertriglyceridemia, 19.8 % had low HDL-C and 87.4 % presented hypertension.

Using the NCEP ATP III criteria, 22.3 % had increased fasting glucose levels, 95.9 %, had increased WC, 84.3 % had hypertriglyceridemia, 22.8 % had decreased HDL-C, and hypertension occurred in 90.9 %.

Using the Chatzi et al. criteria, 47.1 % exhibited high fasting glucose levels, 47.1 % had a pre gestational BMI >30, 91.8 % had hypertriglyceridemia, 37.6 % had low HDL-C and 92.9 % exhibited hypertension.

The Bartha et al. criteria detected the lowest percentage of MetS in pregnancy, with high fasting glucose levels in 83.3 %, pre gestational BMI >30 in 91.7 %, hypertriglyceridemia in 8.3 %, low HDL-C in 16.7 %, and all patients presenting hypertension.

The frequency of the number of MetS components in the NCEP ATP III, harmonized, Bartha and Chatzi criteria are summarized in Table 5. The majority of non-diabetic pregnant women had a cluster of one or two metabolic abnormalities in the four definitions used in this study.

**Table 5:** Frequency of individual components of metabolic syndrome according to four definitions

Defenitions MetS components No	NCEP ATP III [5] N = 675	JIS MetS Criteria [6] N = 675	NCEP ATP III [8,9] N = 675	NHLBI/AHA [8,9] N = 675
0	48	04	239	141
1	200	150	317	252
2	230	274	107	197
3	165	190	12	71
4	32	56	0	14
5	0	1	0	0

Table 6 shows the comparison of socio-demographic and various anthropometric, clinical and biochemical parameters between groups with and without MetS components included in the NCEP ATP III, JIS definition, and modified criteria for MetS in pregnancy by Bartha et al. and Chatzi et al.

**Table 6:** Comparison of socio-demographic, clinical and biochemical parameters in pregnant women with and without metabolic syndrome according to four definitions

Variables	NCEP ATP III [5]		*p* value	JIS MetS Criteria [6]		*p* value
	With MetS N = 197	Without MetS N = 478		With MetS N = 247	Without MetS N = 428	
Mean age (year)	26.2 ± 6.8	24.0 ± 6.6	0.0002	25.6 ± 6.8	24.1 ± 6.7	0.0070
Level of education						
Never studied [n (%)]	21 (10.6)	33 (6.9)	0.1391	24 (9.7)	30 (7.0)	0.2706
Middle	157 (79.7)	415 (86.8)	0.0162	203 (82.2)	369 (86.2)	0.1967
Higher education	19 (9.6)	30 (6.3)	0.1706	20 (8.1)	29 (11.7)	0.6288
Place of residence						
Urban [n (%)]	50 (25.4)	99 (20.7)	0.2196	61 (24.7)	88 (20.6)	0.2495
Village [n (%)]	147 (74.6)	379 (79.3)	0.2196	186 (75.3)	340 (79.4)	0.2495
Current smoker (%)	0 (0.0)	1	1.0	0 (0)	1 (0.2)	1.0
Mean body mass index	–	–	–	–	–	–
Mean waist circumference	97.4 ± 8.5	92.0 ± 9.1	<0.0001	95.6 ± 9.0	92.4 ± 9.3	<0.0001
Known diabetes mellitus (%)	00	00	–	00	00	–
Use of hypotensive drugs (%)	22 (11.1)	22 (4.6)	0.0029	23 (9.3)	21 (4.9)	0.0383
Fasting glycemia (mg/dl)	90.5 ± 29.1	80.9 ± 19.2	<0.0001	91.8 ± 27.4	79.1 ± 18.4	<0.0001
Hipertension	179 (90.9)	191 (39.9)	<0.0001	216 (87.4)	154 (36.0)	<0.0001
Systolic blood pressore	140.8 ± 18.5	124.4 ± 18.0	<0.0001	138.5 ± 18.7	123.8 ± 18.1	<0.0001
Diastolic blood presssure	90.7 ± 14.1	80.4 ± 13.6	<0.0001	89.3 ± 14.3	80.1 ± 13.6	<0.0001
Total cholesterol (mg/dl) [$\overline{x} \pm {\text{s}}$]	197.0 ± 42.9	191.4 ± 35.6	0.0810	196.2 ± 43.6	191.4 ± 33.9	0.1364
LDL-C (mg/dl) [$\overline{x} \pm {\text{s}}$]	122.0 ± 37.8	114.8 ± 32.8	0.020	120.2 ± 38.2	114.9 ± 31.9	0.0655
HDL-C(mg/dl) [$\overline{x} \pm {\text{s}}$]	64.5 ± 19.0	73.5 ± 17.7	<0.0001	65.8 ± 19.1	73.8 ± 17.5	<0.0001
Tryglicerides (mg/dl) [$\overline{x} \pm {\text{s}}$]	182.0 ± 60.2	135.0 ± 47.7	<0.0001	179.7 ± 59.5	131.7 ± 45.1	<0.0001

Variables	NCEP ATP III [10]		*p* value	NHLBI/AHA [8, 9]		*p* value
	With MetS N = 12	Without MetS N = 663		With N = 85	Without N = 590	
Mean age (years)	28.7 ± 2	24.6 ± 6.7	0.0377	25.3 ± 6.9	24.6 ± 6.7	0.3217
Level of education						
Never studied [n (%)]	1 (8.3)	53 (7.9)	1.0	12 (14.1)	42 (7.1)	0.0444
Middle	09 (75)	563 (84.9)	0.588	66 (77.6)	506 (85.8)	0.0744
Higher education	02 (16.7)	47 (7.1)	0.4802	07(8.2)	42 (7.1)	0.8828
Place of residence						
Urban [n (%)]	04 (33.3)	145 (21.9)	0.55	28 (33.0)	121 (20.5)	0.0145
Village [n (%)]	08 (66.7)	518 (78.1)	0.55	57 (67.0)	469 (79.5)	0.0145
Current smoker (%)	1.0	00	1.0	0	1	1.0
Mean body mass index	31.7 ± 4.1	24.2 ± 4.0	<0.0001	27.6 ± 5.0	23.9 ± 3.8	<0.0001
Mean waist circumference	–	–	–	–	–	–
Known diabetes mellitus (%)	00	00	–	00	00	–
Use of hypotensive drugs (%)	3 (25.0)	41 (6.2)	0.0426	11 (12.9)	33 (5.8)	0.0216
Fasting Glycemia (mg/dl)	126.7 ± 46.4	83.0 ± 21.6	0.0076	96.7 ± 34.1	81.9 ± 20.2	0.0002
Hipertension	12(100)	358 (54)	0.0039	79 ± 92.9	291 ± 49.3	<0.0001
Systolic Blood pressore	148.4 ± 20.6	128.8 ± 19.4	0.0006	140.4 ± 17.8	127.6 ± 19.3	<0.0001
Diastolic Blood presssure	96.3 ± 12.4	83.2 ± 14.5	0.00019	90.5 ± 15.6	82.4 ± 14.1	<0.0001
Total cholesterol (mg/dl) [$\overline{x} \pm {\text{s}}$]	174.5 ± 38.1	193.5 ± 37.7	0.043	115.1 ± 36.5	117.1 ± 34.2	0.6155
LDL-C (mg/dl) [$\overline{x} \pm {\text{s}}$]	107.0 ± 34.9	117.0 ± 34.4	0.316	187.8 ± 40.4	193.9 ± 37.4	0.1632
HDL-C (mg/dl) [$\overline{x} \pm {\text{s}}$]	58.0 ± 17.7	71.1 ± 18.5	0.0152	60.0 ± 21.3	72.4 ± 55.3	<0.0001
Tryglicérides (mg/dl) [$\overline{x} \pm {\text{s}}$]	126.7 ± 46.4	83.0 ± 21.6	0.0076	96.7 ± 34.1	81.9 ± 20.2	<0.0001

Non-diabetic pregnant women with MetS were older according to three criteria (NCEP ATP III, the JIS definition and Bartha et al.) and were overweight or obese (either with WC or BMI >30 before pregnancy) according to all four criteria. They had high fasting blood glucose levels, increased serum triglyceride levels, decreased serum HDL-C, increased hypertension prevalence and higher systolic and diastolic blood pressure (p < 0.05).

In the entire population, there were 129 APOs in 95 newborns (14.1 %). The APOs were analyzed according to the four definitions that identified mothers with and without MetS (Table 7). The prevalence of newborns with APOs from non-diabetic mothers with and without MetS defined by the four definitions were similar. There was no statistically significant difference between any APO from mothers with and without MetS. Detailed APO data between the groups with and without MetS mothers are presented in Table 7.

**Table 7:** Comparison of adverse perinatal outcomes in pregnant women with and without metabolic syndrome according to four definitions

Adverse perinatal outcome	Entire study group (N = 675)	NCEP ATP III [5]		p value	JIS MetS Criteria [6]		p value	NCEP ATP III (Bartha [10])		p value	NHLBI/AHA (Chatzi [8, 9]		p value
		With MetS N = 197	Without MetS N = 478		With MetS N = 247	Without MetS N = 428		With MetS N = 12	Without MetS N = 663		With MetS N = 85	Without MetS N = 590	
Visible malformation (%)	1	1 (0.5)	0 (0.0)	0.6468	0 (0)	1 (0.2)	1.0	0 (0.0)	1 (0.2)	1.0	0 (0.0)	1 (0.01)	1.0
Icterus (%)	4	2 (0.1)	2 (0.4)	0.7137	2 (0.8)	2 (0.5)	0.9699	0 (0.0)	4 (0.6)	1.0	1 (1.2)	3 (0.05)	1.0
Macrossomia	28	14 (7.1)	14 (2.9)	0.236	15 (6.0)	13 (3.0)	0.0882	2 (16.7)	26 (3.9)	0.1432	6 (7.0)	22 (3.7)	0.2507
Prematurity (%)	26	7 (3.5)	19 (3.9)	0.9691	9 (3.6)	17 (4.0)	0.9953	1	25 (3.7)	0.9544	5 (5.9)	21 (3.5)	0.4599
Respiratory Distress Syndrome (%)	14	3 (1.5)	11 (2.3)	0.7278	3 (1.2)	11 (2.6)	0.3628	0 (0.0)	14 (2.1)	1.0	2 (2.3)	12 (2.0)	1.0
Stillbirth (%)	15	6 (3.0)	9 (1.9)	0.5192	6 (2.4)	9 (2.1)	0.9952	2 (16.7)	13 (2.0)	0.0148	3 (3.5)	12 (2.0)	0.6305
Apgar score <7													
1 min (%)	31	6 (3.0)	25 (5.2)	0.3028	9 (3.6)	22 (5.1)	0.4816	0 (0.0)	31 (4.8)	0.9433	4 (4.7)	27 (4.6)	1.0
5 min (%)	10	3 (1.5)	7 (1.5)	1.0	3 (1.2)	7 (1.6)	0.9161		10 (1.5)	1.0	1 (1.2)	9 (1.5)	1.0
Number of newborns with APO	95	30 (15.2) (a)	65 (13.6)	0.6658	36 (14.6) (a)	59 (13.8)	0.8655	4 (33.3) (a)	91 (13.7)	0.1293	15 (17.6) (a)	80 (13.5)	0.3973
Any adverse perinatal outcome APO N (%)	129	42 (21.3) (a)	87 (12.2)	0.4069	47 (19.0) (a)	82 (19.2)	1.000	5 (41.7) (a)	124 (18.7)	0.1021	22 (25.9) (a)	106 (18.0)	0.1112

* Difference test of proportions of number of newborns with APO p = 0.3435 (aaa and a). * Difference test of proportions of any adverse perinatal outcomes is p = 0.1898

The agreement and disagreement in the diagnosis of MetS among the four criteria is presented in Table 8. The agreement among these four definitions was ranked from excellent to poor. The agreement was excellent between NCEP ATP III–JIS definition [κ = 0.8332 (0.7894–0.8771), p < 0.001], considerable between Bartha–Chatzi [κ = 0.2232 (0.1172–0.3292), p < 0.001] and Chatzi–JIS definition [κ = 0.4036 (0.3389–0.4684), p < 0.001], moderate between NCEP ATP III–Chatzi [κ = 0.4320 (0.3076–0.5064), p < 0.001] and poor between Bartha–ATP III [κ = 0.0841 (0.0390–0.1293), p < 0.001], and Bartha–JIS definition [κ = 0.0608 (0.00275–0.0941), p < 0.001].

**Table 8:** Agreement and disagreement among metabolic syndrome definitions in pregnant women with metabolic syndrome and the diagnostic criteria according to each definition

Definitions	Concordance			Diagnostic accuracy (%)			
	k-value (95 % CI)	p value	Agreement	Sensitivity	Specificity	PPV	NPV
Bartha vs NCEP ATP III	0.0841 (0.0390–0.1293)	<0.0001	Poor	100	72.1	6.1	100
NCEP ATP III vs Bartha	0.0841 (0.0390–0.1293)	<0.0001	Poor	6.1	100	100	72.1
Bartha vs Chatzi	0.2232 (0.1172–0.3292)	<0.0001	Considerable	100	88.9	14.1	100
Chatzi vs Bartha	0.2232 (0.1172–0.3292)	<0.0001	Considerable	14.1	100	100	88.9
Bartha vs JIS	0.0608 (0.0275–0.0941)	<0.0001	Poor	100	64.6	4.9	100
JIS vs Bartha	0.0608 (0.0275–0.0941)	<0.0001	Poor	4.9	100	100	100
NECP ATP III vs JIS	0.8332 (0.7894–0.8771)	<0.0001	Excellent	100	89.5	79.8	100
JIS vs NECP ATP III	0.8332 (0.7894–0.8771)	<0.0001	Excellent	79.8	100	100	89.5
NCEP ATP III vs Chatzi	0.4320 (0.3076–0.5064)	<0.0001	Moderate	38.1	97.9	88.2	79.3
Chatzi vs NCEP ATP III	0.4320 (0.3076–0.5064)	<0.0001	Moderate	88.2	79.3	38.1	97.9
Chatzi vs JIS	0.4036 (0.3389–0.4684)	<0.0001	Considerable	100	72.5	34.4	100
JIS vs Chatzi	0.4036 (0.3389–0.4684)	<0.0001	Considerable	34.4	100	100	72.5

In terms of the diagnostic accuracy of MetS, the Bartha et al., NCEP ATP III and Chatzi et al. definitions displayed the highest sensitivity (100 %) and negative predictive value (100 %), whereas NCEP ATPIII and the JIS definition had the highest specificity (100 %) and positive predictive value (100 %).

The agreement and disagreement among APO in the four definitions of MetS in non-diabetic pregnant women is presented in Table 9.

**Table 9:** Agreement and disparity among adverse pregnancy outcomes in four definitions of metabolic syndrome in pregnant women

Definitions	Concordance			Diagnostic accuracy (%)			
	k-value (95 % CI)	p value	Agreement	Sensitivity	Specificity	PPV	NPV
Bartha vs NCEP ATP III	0.1739 (0.0214–0.3264)	<0.0001	Slight	100	71.4	13.3	100
NCEP ATP III vs Bartha	0.1739 (0.0214–0.3264)	<0.0001	Slight	13.3	100	100	71.4
Bartha vs Chatzi	0.3798 (0.1095–0.6502)	0.0009	Considerable	100	87.9	26.7	100
Chatzi vs Bartha	0.3798 (0.1095–0.6502)	0.0009	Considerable	26.7	100	100	87.9
Bartha vs JIS	0.1344 (0.0121–0.2567)	<0.0001	Slight	11.1	100	100	64.8
JIS vs Bartha	0.1344 (0.0121–0.2567)	<0.0001	Slight	100	64.8	11.1	100
NCEP ATP III vs Chatzi	0.5215 (0.3364–0.7065)	0.0003	Moderate	93.3	80	46.7	98.5
Chatzi vs NCEP ATP III	0.5215 (0.3364–0.7065)	0.0003	Moderate	46.7	98.5	93.3	80
NECP ATP III vs JIS	0.8613 (0.7549–0.9677)	0.0143	Excellent	83.3	100	100	90.8
JIS vs NECP ATP III	0.8613 (0.7549–0.9677)	0.0143	Excellent	100	90.8	83.3	100
Chatzi vs JIS	0.4701 (0.3005–0.6397)	<0.0001	Moderate	100	73.7	41.7	100
JIS vs Chatzi	0.4701 (0.3005–0.6397)	<0.0001	Moderate	41.7	100	100	73.7

The agreement among these four definitions varies from excellent to poor. The agreement was excellent between NCEP ATP III–JIS definition [κ = 0.86 (0.76–0.9677), p 0.01]; considerable between Bartha–Chatzi [κ = 0.38 (0.11–0.65), p 0.0009]; moderate between NCEP ATP III–Chatzi [κ = 0.52 (0.34–0.71), p 0.003] and Chatzi–JIS definition [κ = 0.47 (0.30–0.64), p < 0.001]; and slight between Bartha–NCEP ATP III [κ = 0.17 (0.02–0.33), p < 0.001] and Bartha–JIS definition [κ = 0.13 (0.01–0.26), p < 0.001].

The Bartha, NCEP ATP III, Chatzi and JIS definitions had the highest sensitivity (100 %) and negative predictive value (100 %), whereas the NCEP ATPIII, JIS definition, and Bartha definitions had the highest specificity (100 %) and positive predictive value (100 %) for APO.

## Discussion

This study described, for the first time, the global variation of MetS prevalence and its components in a sample of non-diabetic pregnant Angolan women, according to four definitions of MetS. Two classical definitions for MetS (NCEP ATP-III and the Joint Interim Statement-JIS) and two other classical definitions modified for pregnant women (Bartha et al. and Chatzi et al.) were evaluated. There are some similarities among the components of the four criteria: none had pre-requisites, three of the five components of MetS are required to establish a MetS diagnosis; and the level for hypertension diagnosis is the same. However, the cutoff for fasting glycemia varies from 100 to 110 mg/dl.

The assessment of obesity is the greatest difference between the four criteria: some use the classical WC, whereas others rely on pre gestational BMI.

Among non-diabetic, black, pregnant Angolan women, the highest prevalence rate was estimated by the Joint Interim Statement (JIS) definition (36.6 %), followed by NCEP ATPIII (29.2 %), Chatzi et al. (12.6 %) and Bartha et al. (1.8 %). Irrespective of the defining criteria, our study revealed a very high prevalence of MetS in non-diabetic, pregnant Angolan women. The overall prevalence (29.2 % by NCEP ATPIII criteria) in pregnant Angolan women is similar to that observed in the Nigerian general population (27.9 %), compared to 34.1 % in the USA, and is higher than the overall prevalence in Angola (17.6 %) and Canada [20].

These results may suggest significant implications for long-term cardiovascular complications, especially in populations of low- to mid-income countries such as Angola. Another explanation for this high prevalence of MetS could be the pregnancy itself, due to the increased abdominal circumference determined either by the gravid uterus or by maternal adaptations to pregnancy as metabolic and hematological changes occur. Many of the parameters involved in the definitions of MetS are part of the maternal adaptations to pregnancy [21].

The significantly different prevalence rates among the four definitions likely arise due to the different criteria used for obesity parameters—WC cut-off points and pre gestational BMI. It is well established in the literature that different components of the criteria impact the MetS prevalence rates and may complicate the interpretation of epidemiological studies [22]. Another aspect that should be considered is the apparent similarity between the normal development of pregnancy to MetS, including a fraction of women showing increased blood glucose levels, higher triglycerides and higher blood pressure levels. GDM and preeclampsia, which are considered features of MetS, are associated with exaggerated dyslipidemia, insulin and glucose metabolism abnormalities during and even after pregnancy. Women with MetS have a higher risk of developing preeclampsia than do those without MetS [23]. An increased prevalence of MetS was found to correspond with the worsening of glucose tolerance in Brazilian hyperglycemic mothers [7].

Pregnancy offers a unique window of time to inform women of the long-term implications of predictive factors such as anthropometric, biochemical and clinical parameters of MetS during pregnancy, which are not only transient metabolic abnormalities but may also be the first manifestation of a serious disease such as Type 2 DM or CVD [7].

The high prevalence of MetS among our patients was surprising because they were low-risk, pregnant women, were at term and had vaginal delivery. This finding indicates that in pregnant women, the criterion used to assess obesity by WC represents a confounding factor because the gravid uterus undermines the measurement. The WC has been proposed as a useful screening tool in many primary care settings, although in pregnant women, the WC is potentially problematic because the uterine size could interfere in this measurement. This has led to some confusion on the part of obstetricians regarding how to identify pregnant women with MetS. During pregnancy, the characterization of central obesity is difficult due to uterus enlargement. The suggested WC cut-off points to define MetS result from experts’ consensus and, in our view, justify additional clinical and epidemiological prospective studies on pregnant women by using adverse perinatal outcomes as a dependent variable.

Recent guidelines released by the Joint Interim Statement (JIS) [6] stressed the need to adopt ethnicity-specific values of WC to measure central obesity. For a given WC, Asians, blacks, and Caucasians showed different levels of intra-abdominal adiposity, thus putting the subjects at different levels of risk for CVD and type2 DM [24, 25].

However, these guidelines did not determine how to evaluate MetS during pregnancy, which cut-off points should be used to define abdominal obesity, the prevalence of MetS during pregnancy worldwide, and the impact of MetS both in pregnancy and in the perinatal period. The various diagnostic criteria have led to some confusion on the part of clinicians regarding how to identify pregnant women with MetS.

This issue suggests the need to reconsider the current cut-off points for WC to establish specific recommendations for pregnant women.

Although the two classical definitions of MetS were introduced in 2001 and 2005 and the consensus statement was established in 2009 [2], disagreement remains regarding their use in clinical practice. This debate is caused by disparities in the results, as evidenced by the review by Oguoma, VM 2014 in Prevalence of cardio-metabolic syndrome in Nigeria: a systematic review [20].

Conversely, the two definitions that have modified the classical consensus for MetS in pregnancy detected the lowest levels of MetS in non-diabetic, pregnant Angolan women [Chatzi et al. (12.6 %) and Bartha et al. (1.8 %)]. The definition proposed by Chatzi was based on an adaptation of the NHLB/AHA criteria, and obesity is defined as a pre gestational BMI >30 kg/m^2^. The authors analyzed the association between MetS characteristics before pregnancy and early in pregnancy and concluded that women with these MetS components were at a high risk of GDM development. The definition of obesity according to the WHO criteria (BMI > 30 kg/m^2^) was used in four other studies evaluating MetS in pregnant women (Chatzi et al. preterm; Bartha et al.). These studies showed that MetS is not only directly responsible for the development of atherosclerotic cardiovascular disease but that it has additional impacts on human pregnancy, such as preterm delivery, low birth weight infants, and the development of diseases such as diabetes, preeclampsia and hypertension.

Considering the apparent disagreement surrounding the evaluation of central obesity in the diagnosis of MetS, we found a substantial degree of agreement between the JIS definition and the NCEP ATPIII (0.83), which indicates that the requirement of abdominal obesity did not generate significant discrepancies in the prevalence or the classification of the MetS; instead, the WC cut-off points did. Similar results were found in a large Chinese population, other Caucasian populations [27, 28], and in Luxembourg [28].

The definitions using pre gestational BMI in pregnancy exhibited considerable (0.22) agreement.

African pregnant women with MetS were older according to three criteria (NCEP ATP III, the JIS definition and Bartha et al.) and more overweight or obese either with the WC or BMI >30 before pregnancy variables based on all four criteria. They had higher fasting glycemia, increased serum triglyceride levels, decreased serum HDL-C, increased hypertension prevalence and higher systolic and diastolic blood pressure.

Among the four definitions used, the JIS definition was found to be the most sensitive, and the NCEP ATPIII definition was found to be most specific in identifying cases of MetS.

APO represents a group of maternal diseases and/or neonatal morbidity that interfere in perinatal results. It is well known that perinatal outcomes and quality of prenatal care are separate but related issues. Furthermore, perinatal outcomes depend in part upon the quality of care, the complexity of the case mix and maternal complications. In a low-risk pregnant population from Africa, the prevalence of APO (14.1 %) was similar in mothers with and without MetS based on all four definitions. These results conflict with those found by Negrato et al. in a Brazilian cohort of hyperglycemic mothers who had or did not have MetS. The offspring of the hyperglycemic mothers with MetS presented significantly higher prevalences of Large for Gestational Age (LGA), overweight (ponderal index), Apgar scores (<7 at 1 min and 2 min) and any type of APOs.

Our study had a cross-sectional design and was conducted among low-risk pregnant women from Huambo Hospital, which is located in the central highlands of Angola, a sub-Saharan area in Africa that does not necessarily represent the entire pregnant population of Angola.

However, several points of strength do need to be highlighted. First is the high prevalence of MetS according to four definition criteria, considering the increased risk of future CVD diseases among non-diabetic, pregnant Angolan women. Second, this study describes the similarities and differences among the classical definitions for MetS currently used outside pregnancy and those used in pregnancy to establish maternal and perinatal risks of MetS. This adverse maternal metabolic profile contributes to the epidemiological mapping of MetS in Angola and can serve as baseline data for low-risk pregnant women.

The completion of the JIS meeting to harmonize the criteria for the diagnosis of MetS emphasize that the new diagnosis elements, especially the WC, need to be addressed in the near future (JIS 2009). In our view, it is also necessary to establish correct and harmonized criteria of MetS in pregnancy, which can be accomplished by longitudinal studies comparing pregnant women who are at low and high risk for MetS.

Our results show the importance of strategic efforts to improve prenatal care to avoid maternal and perinatal long-term consequences. This includes focusing on interventions such as whole family lifestyle modification with weight control, physical activity, and nutrition to control the MetS epidemic of the 21st century both during and after pregnancy.

## Conclusions

The prevalence of metabolic syndrome in nondiabetic pregnant women considered criteria adapted pregnancy by Chatzi et al. (12.6 %) by Bartha et al. (1.8 %). Perinatal adverse outcome were higher in infants of mothers with metabolic syndrome, 41.7 % for criteria Bartha et al., and 25.9 % for the criteria Chatzi et al. This explains once again that women with metabolic syndrome have higher risk for newborns with perinatal adverse outcome, calling attention to the need to prevent pre gestational syndrome, control it during pregnancy, especially in the first half.

We think that the criteria that best fits for the diagnosis of metabolic syndrome in pregnancy are the Bartha et al., because women experience during pregnancy so forwarding components of metabolic syndrome. Bartha et al. to consider these transformations, defined best cut-off points so that better predicts adverse perinatal outcomes.

## Abbreviations

MetS: metabolic syndrome
DM: diabetes mellitus
CVD: cardiovascular disease
IR: insulin resistance
HDL: higher-density lipoproteins
WHO: World Half Organization
NCEP ATP III: National Cholesterol Education Program Third Adult Treatment Panel III
JIS: joint interim statement
APO: adverse perinatal outcomes
GDM: gestational diabetes mellitus
BMI: body mean index
SD: standard deviation
WC: waist circumference
HNLBI/AHA: National Heart, Lung, and Blood Institute/American Heart

## References

[1] Reaven GM (1988). Banting lecture, role of insulin resistance in human disease. Diabetes.

[2] Kassi E, Pervanidou P, Kaltsas G, Chrousos G (2011). Metabolic syndrome: definitions and controversies. BMC Med.

[3] Alberti KGMM, Zimmet PZ (1998). Definition, diagnosis and classification of diabetes mellitus and its complications. Part 1: diagnosis and classification of diabetes mellitus provisional report of a WHO consultation. Diabet Med.

[4] OMS Organização Mundial da Saúde (1999). WHO consultation: definition, diagnosis and classification of diabetes mellitus and complications. WHO/NCD/NCS.

[5] Expert Panel on Detection (2001). Evaluation, and treatment of high blood cholesterol in adults. Executive summary of the third report of the national cholesterol education program expert panel on detection, evaluation, and treatment of high blood cholesterol in adults (adult treatment panel III). JAMA.

[6] Alberti KGMM, Eckel RH, Grundy SM, Zimmet PZ, Cleeman JI, Donato KA (2009). Harmonizing the metabolic syndrome: a joint interim statement of the International Diabetes Federation task force on epidemiology and prevention; National Heart, Lung, and Blood Institute; American Heart Association; World Heart Federation; International Atherosclerosis Society; and International Association for the Study of Obesity. Circulation.

[7] Negrato CA, Jovanovic L, Alex R, Tambascia MA, Dias A, Rudge MVC (2009). Association between different levels of dysglycemia and metabolic syndrome in pregnancy. Diabetol Metab Syndr..

[8] Chatzi L, Plana E, Daraki V, Karakosta P, Alegkakis D, Tsatsanis C, Kafatos C, Koutis A, Kogevinas M (2009). Metabolic syndrome in early pregnancy and risk of preterm birth. Am J Epidemiol.

[9] Chatzi L, Plana E, Pappas A, Alegkakis D, Karakosta P, Daraki V, Vassilaki M, Tsatsanis C, Koutis A, Kogevinas M (2009). The metabolic syndrome in early pregnancy and risk of gestational diabetes mellitus. Diabetes Metab J.

[10] Bartha JL, Bugatto FG, Macías RF, González LN, Delgado RC, Vivancos BH (2008). Metabolic Syndrome in normal and complicated pregnancies. Eur J Obstet Gynecol Reprod Biol.

[11] Mann S, Pratt S, Gluck P, Nielsen P, Risser D, Greenberg P, Marcus R (2006). Assessing quality obstetrical care: development of standardized measures. Jt Comm J Qual Patient Safety.

[12] Nielsen PE, Goldman MB, Mann S, Shapiro DE, Marcus RG, Pratt SD, Greenberg P (2007). Effects of teamwork training on adverse outcomes and process of care in labor and delivery: a randomized controlled trial. Obstet Gynecol.

[13] Pettker CM, Thung SF, Norwitz ER, Buhimschi CS, Raab CA, Copel JA, Kuczynski E (2005). Impact of a comprehensive patient safety strategy on obstetric adverse events. Am J Obstet Gynecol.

[14] Hamilton E, Smith S, Berry D, Dfazhrm RN, Bem O, Keeffe DO, Knox E. Revisting the perinatal adverse outcame index. Patient Safety Quality Healthcare. 2011.

[15] Magalhães P, Capingana DP, Mill JG (2014). Prevalence of the metabolic syndrome and determination of optimal cut-off values of waist circumference in university employees from Angola. Cardiovasc J Afr..

[16] Topouchian JA, El Assaad MA, Orobinskaia LV, El Feghali RN, Asmar RG (2005). Validation of two devices for self-measurement of brachial blood pressure according to the International Protocol of the European Society of Hypertension: the SEINEX SE-9400 and the Microlife BP 3AC1-1. Blood Press Monit..

[17] Battaglia FC, Lubchenco LO (1967). A practical classification of newborn infants by weight and gestational age. J Pediatr.

[18] Landis JR, Koch GG (1977). The measurement of observer agreement for categorical data. Biometrics..

[19] Oguoma VM, Nwose BC, Richards RS (2015). Prevalence of cardio-metabolic syndrome in Nigeria: a systematic review. Public Health..

[20] Willians, 23ª Edição, Cunningham FG, Kenneth J. Leveno, MD, Bloom LS, Hauth JC, Rouse JD, Spong YC. Williams obstetrics, 23rd edn. 2014. p 108–114.

[21] Hollman G, Kristenson M (2008). The prevalence of the metabolic syndrome and its risk factors in a middle-aged Swedish population–mainly a function of overweight?. Eur J Cardiovasc Nurs.

[22] Yang JJ, Lee SA, Choi JY, Song M, Han S, Yoon HS, Lee Y, Juhwan OH, Lee JK, Kang D (2015). Subsequent risk of metabolic syndrome in women with a history of preeclampsia: data from the health examinees study. J Epidemiol..

[23] Ford ES, Li C, Zhao G (2010). Prevalence and correlates of metabolic syndrome based on a harmonious definition among adults in the US. J Diabetes.

[24] Athyros VG, Ganotakis ES, Tziomalos K, Papageorgiou AA, Anagnostis P, Griva T, Kargiotis K, Mitsiou EK, Karagiannis A, Mikhailidis DP (2010). Comparison of four definitions of the metabolic syndrome in a Greek (mediterranean) population. Curr Med Res Opin.

[25] Misra A, Khurana L (2008). Obesity and the metabolic syndrome in developing countries. J Clin Endocrinol Metab.

[26] Diamanti-Kandarakis E, Papavassiliou AG, Kandarakis SA, Chrousos GP (2007). Pathophysiology and types of dyslipidemia in PCOS. Trends Endocrinol Metab.

[27] World Health Organisation (2000). Non-communicable diseases: a strategy for the African region.

[28] Alkerwi A, Donneau AF, Sauvageot N, Lair ML, Scheen A, Albert A, Guillaume M (2011). Prevalence of the metabolic syndrome in Luxembourg according to the Joint Interim Statement definition estimated from the ORISCAV-LUX study. BMC Public Health..

